# Transcriptional Response and Morphological Features of the Neurovascular Unit and Associated Extracellular Matrix After Experimental Stroke in Mice

**DOI:** 10.1007/s12035-019-1604-4

**Published:** 2019-05-14

**Authors:** Susanne Aleithe, Alexandra Blietz, Bianca Mages, Constance Hobusch, Wolfgang Härtig, Dominik Michalski

**Affiliations:** 1 Department of Neurology, University of Leipzig, Liebigstr. 20, 04103 Leipzig, Germany; 2 University of Leipzig, Liebigstr. 19, 04103 Leipzig, Germany; 3 Institute of Anatomy, University of Leipzig, Liebigstr. 13, 04103 Leipzig, Germany

**Keywords:** Stroke, Focal cerebral ischemia, Gene expression, Neurovascular unit, Extracellular matrix

## Abstract

Experimental stroke studies yielded insights into single reactions of the neurovascular unit (NVU) and associated extracellular matrix (ECM). However, the extent of simultaneous processes caused by ischemia and their underlying transcriptional changes are still poorly understood. Strictly following the NVU and ECM concept, this study explored transcriptional responses of cellular and non-cellular components as well as their morphological characteristics following ischemia. Mice were subjected to 4 or 24 h of unilateral middle cerebral artery occlusion. In the neocortex and the striatum, cytoskeletal and glial elements as well as blood-brain barrier and ECM components were analyzed using real-time PCR. Western blot analyses allowed characterization of protein levels and multiple immunofluorescence labeling enabled morphological assessment. Out of 37 genes analyzed, the majority exhibited decreased mRNA levels in ischemic areas, while changes occurred as early as 4 h after ischemia. Down-regulated mRNA levels were predominantly localized in the neocortex, such as the structural elements α-catenin 2, N-cadherin, β-catenin 1, and βIII-tubulin, consistently decreasing 4 and 24 h after ischemia. However, a few genes, e.g., claudin-5 and Pcam1, exhibited increased mRNA levels after ischemia. For several components such as βIII-tubulin, N-cadherin, and β-catenin 1, matching transcriptional and immunofluorescence signals were obtained, whereas a few markers including neurofilaments exhibited opposite directions. In conclusion, the variety in gene regulation emphasizes the complexity of interactions within the ischemia-affected NVU and ECM. These data might help to focus future research on a set of highly sensitive elements, which might prospectively facilitate neuroprotective strategies beyond the traditional single target perspective.

## Introduction

Along with an increasing life expectancy of the population in most countries, the rate of age-related diseases, i.e., cardiovascular diseases including stroke, will probably increase during the next decades [1]. Stroke ranged among the five most frequent causes of death worldwide and accounts for one of the most common reasons of long-lasting disability [2, 3]. In recent years, stroke treatment has substantially improved, especially by adding techniques for re-opening of occluded cerebral vessels [4, 5]. However, since a substantial proportion of patients is still not eligible for these treatments [6], an ongoing need exists for the development of novel neuroprotective strategies.

As a cellular-based perspective of stroke-related tissue damage, the “neurovascular unit” (NVU) was conceptualized to summarize the network of brain cells affected by the ischemic event [7, 8]. The NVU classically comprises glial elements such as microglia and astrocytes, and the vasculature with its endothelium as well as neurons themselves [9]. Recently, ischemia-caused cellular alterations were also described for oligodendrocytes [10], resulting in an extended NVU concept also involving oligodendroglial structures. Functionally, elements of the NVU—especially endothelial cells involved in forming the blood-brain barrier (BBB)—regulate the influx of water and blood-borne molecules into the parenchyma [11]. Under ischemic conditions, a significant impairment of the BBB integrity is associated with diverse morphological alterations of cellular NVU components and consecutive edema formation [12, 13]. In addition to cellular consequences, ischemic stroke was found to affect the extracellular matrix (ECM) and its perineuronal nets (PNs) [14–17], which are assumed to modulate cellular integrity and neural plasticity [18–20]. Basically, PNs are formed by several components such as chondroitin sulfate proteoglycans (CSPGs) and their glycosaminoglycans, hyaluronic acid, and tenascins, known to build polyanionic lattice-like coatings of certain neurons [21–25]. Apart from the ECM, the cytoskeleton is supposed to play a crucial role in cellular integrity within the NVU [26]. Here, neurofilaments together with elastic and fibrous proteins as well as microtubules and actin filaments are regularly formed as a highly interactive network [26–28]. Further, these structures are complemented by various transmembrane cell adhesion molecules, which allow inter-endothelial cell-cell contacts or interactions with elements beyond the perivascular space [29, 30].

The large number of potentially involved cell-cell and cell-matrix interactions illustrates the complexity within the NVU and associated ECM. To pave the way for the development of new treatment strategies, a better understanding of pathophysiological processes is essential. Addressing this issue, previous studies utilized histochemical techniques [14, 31, 32], electron microscopy [e.g., 13] as well as gene expression analyses by northern blotting techniques [33] to explore changes within the NVU caused by experimental stroke. However, these techniques generally harbor the limitation that only a small number of targeted elements originating from the same tissue can be explored at the same time, which drastically limits the interpretation of a single finding. As one approach to overcome this issue, studies applying gene expression profiling have been used to focus on post-ischemic consequences to the brain [34–38]. Notably, as these investigations predominantly used peripheral blood probes, direct conclusions regarding local brain alterations became difficult, resulting in the situation that no satisfactorily stable biomarker—originating from the brain itself—is so far applicable for the clinical situation [39]. Subsequent efforts included combined analyses of human blood samples and ischemia-affected rodent brains which revealed altered messenger RNA (mRNA) expression [40]. In parallel, studies involving animals detected the genomic response under various ischemic conditions and addressed processes like inflammation, apoptosis, and neuroprotection [41–44], while other studies used widespread mRNA level microarrays [39–41, 45, 46]. However, only a few approaches explored ischemic consequences to certain gene levels by quantitative real-time polymerase chain reaction (qRT-PCR) [34, 45, 46] extracted from specified brain areas, which would technically allow a correlation with morphological features of the ischemic tissue and thus help to interpret findings in terms of altered mRNA levels.

Applying a combined biochemical and histochemical approach, this study aimed to explore simultaneous reactions of cellular and non-cellular elements of the ischemia-affected NVU and associated ECM. Analyses were primary focused on the transcriptional response in terms of mRNA levels from various genes, followed by morphological characterizations as captured by multiple immunofluorescence labeling. To consider time-dependent features of stroke [47], analyses included two different durations of ischemia, i.e., 4 and 24 h.

## Materials and Methods

### Study Design

Multiple fluorescence labeling as well as qRT-PCR and Western blot analyses were applied to characterize ischemia-affected brains from a total of 20 adult male C57BL/6J mice with a mean body weight of 25 g, provided by Charles River (Sulzfeld, Germany). Experimental focal cerebral ischemia was achieved by a unilateral filament-based model resulting in right-sided middle cerebral artery occlusion (details given below). Neurobehavioral deficits were assessed by the Menzies Score [48], ranging from 0 (no neuronal deficit) to 4 (spontaneous contralateral circling). To confirm adequate stroke induction, mice had to demonstrate at least a score of 2, serving as a pre-defined study inclusion criterion. Overall, brains from 10 animals were used for sample recovery used for qRT-PCR and Western blot analyses (*n* = 5 for 4 and 24 h each), and brains from 10 animal served for immunohistochemical analyses (*n* = 5 for 4 and 24 h each). Techniques for tissue characterization addressed the neocortex and striatum of the ischemia-affected hemisphere and the corresponding contralateral regions for control purposes.

Generally, animals had free access to food and water throughout the whole study. Animal experiments were performed according to the European Union Directive 2010/63/EU and the German guideline for the use of laboratory animals after approval by local authorities (Regierungspräsidium Leipzig, reference number TVV 02/17). Reporting of animal experiments followed the ARRIVE guidelines.

### Experimental Focal Cerebral Ischemia

Unilateral middle cerebral artery occlusion was performed as originally described by Longa et al. [49] with some minor modifications as formerly reported [50]. For surgical procedures, mice were anesthetized using 2–2.5% isoflurane (Baxter, Unterschleißheim, Germany) and a vaporisator (VIP 3000, Matrix, New York, USA) with a mixture of 70% N_2_O/30% O_2_. After preparation of the right-sided cervical vessels, a standardized silicon-coated 6-0 monofilament (Doccol Corporation, Redlands, CA, USA) was introduced into the internal carotid artery. The filament was carefully pushed forward to the origin of the right middle cerebral artery as indicated by bending observed or resistance felt. During the intervention, the body temperature of the mice was controlled and adjusted to 37 °C using a rectal probe and a thermostatically regulated warming pad (Fine Science Tools, Heidelberg, Germany). After 4 or 24 h, mice were sacrificed for subsequent histochemical and molecular biological analyses.

### Tissue Preparation

Mice ascribed to qRT-PCR and Western blot analyses were perfused with saline only. Subsequently, brains were extracted and areas of the ischemia-affected hemisphere were identified by the ischemia-related edema and then manually dissected into pieces, comprising either neocortex or striatum, same as the respective control areas of the contralateral hemisphere. Finally, all brain samples were flash-frozen in liquid nitrogen and stored at - 80 °C. For histochemical analyses, mice were transcardially perfused with saline and 4% paraformaldehyde (PFA) in 0.1 M phosphate-buffered saline, pH 7.4 (PBS). Brains were then removed from the skulls and post-fixed in the same fixative for 24 h, followed by equilibration in 30% phosphate-buffered sucrose. Afterwards, the forebrains were serially cut into 30-μm-thick coronal sections by using a freezing microtome (Leica SM 2000R, Leica Biosystems, Wetzlar, Germany). All brain sections were stored at 4 °C in 0.1 M Tris-buffered saline, pH 7.4 (TBS) containing 0.2% sodium azide prior to histochemical procedures.

### RNA Isolation and Quantitative Real-Time PCR

Total RNA was extracted according to the peqGOLD RNAPure™ manual for lipid-rich tissues (VWR, Darmstadt, Germany) and the obtained RNA concentrations were quantified by using a NanoDrop (VWR). Five hundred nanograms of RNA was reverse-transcribed with a mixture of oligo(dT) and random primers and the Proto Script M-MuLV First Strand cDNA Synthesis Kit (New England Biolabs, Frankfurt/Main, Germany). The resulting cDNA samples were then quantified for each test gene by target gene-specific, intron-spanning primer pairs designed using the online tool Primer3web version 4.1.0 (http://primer3.ut.ee/) and utilized from Biomers (Ulm, Germany). Primers considered all splice variants (SpV) from genes of interest (Table 1). For qRT-PCR measurements, the levels of mRNA transcripts were quantified by using the RotorGene 2000 (Qiagen, Hilden, Germany) and the conformed Rotor-Gene SYBR Green PCR Kit (Qiagen), also according to the manufacturer’s standard protocol. Expression levels of the genes of interest and of the respective housekeeping genes were detected based on individual gene standard curve equations, which were calculated by plotting the log10 of corresponding target dilutions on the X-axis against the threshold cycle (Ct) value from a dilution series of target DNA on the Y-axis. Each sample was analyzed in repeat determination. For normalization of each sample, β-actin (*Actb*) and glyceraldehyde 3-phosphate dehydrogenase (*Gapdh*) (data on the latter one not shown) were used as reference genes [51, 52]. Fold changes of distinct genes were used to capture ischemia-related changes of mRNA levels on the ipsilateral, i.e., ischemia-affected hemisphere based on an inter-hemispheric comparison, thereby using the contralateral, i.e., non-affected hemisphere as control region on the individual level (fold change = ([gene of interest/housekeeping gene] _ipsilateral_ / [gene of interest/housekeeping gene] _contralateral_)).

**Table 1:** Primer used for qRT-PCR analyses

Category	Genes	Reference-sequence	Forward primer	Reverse primer
Structural elements	*Cdh2*	NM_007664.5	TTACCTCAAGAGGCGGAGAC	GCAGGATGGAAATGTTGGAC
	*Cdh5*	NM_009868.4	CACTGCTTTGGGAGCCTTC	GGCAGCGATTCATTTTTCTC
	*Ctnna2*	NM_145732.2	CTCACTGAGGCAGTGGATGA	GTGGCTTCCAGCACTTTCTC
	*Ctnnb1*	NM_007614.3	GCAGCAGCAGTCTTACTTGG	CCCTCATCTAGCGTCTCAGG
	*Tubb3*	NM_023279.2	CCAGCGGCAACTATGTAGG	AAGTTGTCGGGCCTGAATAG
Neurofilaments	*Ina*	NM_146100.4	AAATGGCCCTTGACATTGAG	TGGGAGGGAGCAAATAACTG
	*Nefh*	NM_010904.3	ACTCTCAGAGGCAGCCAAAG	AGCAGGTCCTGGTACTCTCG
	*Nefl*	NM_010910.1	TCAAGGCTAAGACCCTGGAG	AGGCCATCTTGACATTGAGG
	*Nefm*	NM_008691.2	AAACTCCTAGAGGGGGAAGA	GCCTCGACTTTGGTCTTCTG
Blood-brain barrier	*Cldn1*	NM_016674.4	TGATCGCAATCTTTGTGTCC	GCTGTGGCCACTAATGTCG
	*Cldn3*	NM_009902.4	CCAACTGCGTACAAGACGAG	TACAACCCAGCTCCCATCTC
	*Cldn5*	NM_013805.4	CTGGACCACAACATCGTGAC	AGTGCTACCCGTGCCTTAAC
	*F11r*	NM_172647.2	TGCTTACAGCAGATGCCAAG	TGGGCCTGGCAGTAGTATTC
	*Ocln*	NM_008756.2	CCTGGGGTTCATGATTATCG	TTTGCCATTGGAGGAGTAGG
	*Pecam1*	NM_008816.3	CGATGCGATGGTGTATAACG	CCATGAGCACAAAGTTCTCG
	*Tjp1*	NM_009386.2	TTCACACCAAAGCCGTACAC	TCACAGGGACAGCTTTAGGC
Glial markers	*Aif1*	NM_019467.2	GATGCCTGGGAGTTAGCAAG	AGACGCTGGTTGTCTTAGGC
	*Gfap*	NM_010277.3	GCACTCAATACGAGGCAGTG	CGGCGATAGTCGTTAGCTTC
	*Glul*	NM_008131.3	CAGGGTGAGAAAGTCCAAGC	AGCTTGTTGGGGTCTTTGC
	*S100b*	NM_009115.3	AGAGGGTGACAAGCACAAGC	TCCATCACTTTGTCCACCAC
Extracellular matrix	*Has1*	NM_008215.2	AACGTGAGGTCATGTACACAGC	AGCTCTGACAAGCTCGTTCC
	*Has2*	NM_008216.3	GGGACCTGGTGAGACAGAAG	TCTCCTCCAACACCTCCAAC
	*Itga5*	NM_010577.4	GCCTGAAGCTGTGATTTTCC	TGCTGAGTCCTGTCACCTTG
	*Itgb8*	NM_177290.3	AAACTTGCAAGCCACAGGAG	ATCTGCCACCTTCACACTCC
	*Thsb1*	NM_011580.4	GCTATCTGTGGCCTCTCCTG	TTCAGCTCACTGACCAGCTC
	*Thsb2*	NM_011581.3	ATGTGGGCTGCGATCTTATC	TCTGCAAACACGAGATGGAC
Perineuronal nets	*Acan*	NM_007424.2	GCCCTTCACGTGTAAAAAGG	CAGGTGATTCGAGGCTCTTC
	*Bcan*	NM_007529.2	ATTGGGCTCAATGACAGGAC	CCCCAGACAGGAAGTAGCTG
	*Csgalnatc1*	NM_172753.5	CTGAGCTGAACACGGTGCTA	TCTGCTTCTGGTGGTGACTG
	*Ncan*	NM_007789.3	ATGGTTTCATCTGCCTCTGC	AGCAGTGTCCCTGGAATTTG
	*Ptprz1*	NM_001311064.1	CGTCCTTGGAAAACACGTTC	TCCAGTGGGAACTTCTGTCC
	*Vcan (SpV1)*	NM_019389.2	CCTCACAAGCATCCTTTCTCA	CAGCGGAAGTCATGTTCAAA
	*Vcan (SpV2)*	NM_019389.2	AGCCCTTTCTCACAGCTCAG	TTGAGCACATCCATAAGATGC
Net-bearing neurons	*Gad1*	NM_008077.5	ACTGGGCCTGAAGATCTGTG	GGAGAAGTCGGTCTCTGTGC
	*Gad2*	NM_008078.2	CGATTTCCATTACCCCAATG	CAACCAGTCTGCTGCTAATCC
	*Kcnc1*	NM_008421.3	GTTCGAGGACCCCTACTCATC	TTTCGGTCTTGTTCACGATG
	*Pvalb*	NM_013645.4	GGATGTCGATGACAGACGTG	TCCGGGTTCTTTTTCTTCAG
Housekeeping genes	*Actb*	NM_007393.3	CATCCGTAAAGACCTCTATGCCAAC	ATGGAGCCACCGATCCACA
	*Gapdh*	NM_008084.3	GCCAAGGCTGTGGGCAAGGT	TCTCCAGGCGGCACGTCAGA

*SpV1/SpV2* splice variant 1/splice variant 2

### Western Blot Analyses

For Western blot analyses For Western blot analyses, (reagents are listed in Table 2), total proteins were extracted, homogenized, and lysed by ultrasonification of the brain samples in 60 mM Tris-HCl, pH 6.8, containing 2% sodium dodecyl sulfate (SDS) and 10% sucrose, supplemented with a protease inhibitor cocktail (Cell Signaling, Leiden, The Netherlands), followed by centrifugation for 10 min (13,000 rpm; 4 °C). Measuring of protein concentrations was performed by using a BCA kit (Thermo Fisher, Waltham, MA, USA). Subsequently, proteins were denaturated in sample buffer (250 mM Tris-HCl, pH 6.8, containing 4% SDS, 10% glycerol, and 2% β-mercaptoethanol) at 95 °C for 5 min. Proteins were separated using a 7.5% SDS-PAGE for Pecam1 and 10% SDS-PAGE for Has1 and converted to nitrocellulose membranes (Th.Geyer, Renningen, Germany). Afterwards, membranes were blocked with 5% dry milk in TBS (50 mM Tris-HCl, 150 mM NaCl, pH 7.5) for 30 min and incubated with primary antibodies (Table 2) at 4 °C overnight. After washing the membranes three times with 0.1% *v*/*v* Tween in Tris-buffered saline (TBST), membranes were incubated with the corresponding horseradish peroxidase (HRP)-conjugated secondary antibodies (Table 2) and target proteins were detected using enhanced chemiluminescence (ECL) according to the manufacturer’s (ThemoFisher) instructions. After image acquisition, membranes were stripped with stripping buffer (15 g/l glycine, 1 g/l SDS, 10 ml/l Tween 20, pH 2.2) and reused to detect β-actin as an internal protein reference to calculate the relative protein concentration.

**Table 2:** Antibodies used for Western blotting

Primary antibodies	Secondary antibodies
rabbit-anti-HAS1 (1:1000; Thermo Fisher, Waltham, MA, USA)	HRP-horse-anti-rabbit IgG (1:10,000; Vector Labs, Burlingame, CA, USA)
rabbit-anti-CD31 (1:1000; EPRI17260-265; Abcam; Cambridge, England)	HRP-horse-anti-rabbit IgG (1:10,000; Vector Labs)
mouse-anti-β-actin (1:2000; Cell Signaling, Danvers, MA, USA)	HRP-horse-anti-mouse IgG (1:10,000; Vector Labs)

### Fluorescence Labeling and Microscopy

For fluorescence labeling, tissues were extensively rinsed in TBS and their non-specific binding sites were subsequently blocked with TBS containing 5% normal donkey serum and 0.3% Triton X-100 for 1 h. Afterwards, the sections were incubated with primary antibodies (diluted in the blocking solution) for 20 h, then washed again with TBS followed by incubation in mixtures of fluorochromated secondary antibodies for 1 h (immunoreagents listed in Table 3). Thereafter, the sections were thoroughly rinsed with TBS, briefly washed with distilled water, mounted onto fluorescence-free glass slides, and cover-slipped with Entellan in toluene (Merck, Darmstadt, Germany). The omission of primary antibodies resulted in the absence of any labeling (Table 2). Fluorescence images were captured with a Biorevo BZ-9000 microscope (Keyence, Neu-Isenburg, Germany).

**Table 3:** Immunoreagents used for triple fluorescence labeling

First marker	First marker	Second marker	Second marker	Third marker	Third marker
Primary antibodies	Secondary reagents^a^	Primary antibodies	Secondary reagents^a^	Primary antibodies	Secondary reagents^a^
rabbit-anti-Nefl (1:200; Synaptic Systems, Göttingen, Germany)	Cy3-donkey-anti-rabbit IgG	guinea pig-anti-Gfap (1:200; Synaptic Systems)	Cy2-donkey-anti-guinea pig IgG	biotinylated mouse-anti-NeuN (1:50; Merck Millipore; Billerica, MD; USA)	Cy5-streptavidin
rabbit-anti-Nefl (1:200; Synaptic Systems)	Cy3-donkey-anti-rabbit IgG	guinea pig-anti-Iba (1:100; Synaptic Systems)	Cy2-donkey-anti-guinea pig IgG	biotinylated mouse-anti-CD31 (1:25; Serotec; Puchheim, Germany)	Cy5-streptavidin
rabbit-anti-N-Cadherin (1:500; Synaptic Systems)	Cy3-donkey-anti-rabbit IgG	guinea pig-anti-βIII-Tubulin (1:200; Synaptic Systems)	Cy2-donkey-anti-guinea pig IgG	biotinylated mouse-anti-NeuN (1:50; Merck Millipore)	Cy5-streptavidin
rabbit-anti-β-Catenin (1:500; Synaptic Systems)	Cy3-donkey-anti-rabbit IgG	guinea pig-anti-βIII-Tubulin (1:200; Synaptic Systems)	Cy2-donkey-anti-guinea pig IgG	biotinylated mouse-anti-NeuN (1:50; Merck Millipore)	Cy5-streptavidin
rabbit-anti-S100β (1:600; Synaptic Systems)	Cy3-donkey-anti-rabbit IgG	guinea pig-anti-Gfap (1:400; Synaptic Systems)	Cy2-donkey-anti-guinea pig IgG	mouse-anti-GS (1:100; Merck Millipore)	Cy5-donkey-anti-mouse IgG
biotinylated WFA (15 μg/ml; Vector Laboratories; Burlingame, CA, USA)	Cy3-streptavidin	rabbit-anti-Gad1 (1:200; Synaptic Systems)	Cy2-donkey-anti-rabbit IgG	guinea pig-anti-Pvalb (1:300; Synaptic Systems)	Cy5-donkey-anti-guinea pig IgG
mouse-anti-Acan (1:200; Acris Antibodies GmbH, Herford, Germany)	Cy3-donkey-anti-mouse IgG	guinea pig-anti-Pvalb (1:300; Synaptic Systems)	Cy2-donkey-anti-guinea pig IgG	rabbit-anti-Kv3.1b (1:1000; [53])	Cy5-donkey-anti-rabbit IgG

Abbreviations: *CD31* cluster of differentiation 31 (also known as Pecam1 = Platelet endothelial cell adhesion molecule), *Iba* ionized calcium-binding adapter molecule 1 (also known as Aif1 = Allograft inflammatory factor 1), *GS* glutamine synthetase (also known as Glul), *Kv3.1b* Potassium voltage-gated channel subfamily C member 1 (also known as Kcnc1), *WFA Wisteria floribunda* agglutinin

^a^All secondary immunoreagents were obtained from Dianova (Hamburg, Germany) as supplier for Jackson ImmunoResearch (West Grove, PA, USA)

### Statistical Analyses and Image Processing

All data were processed with GraphPad Prism (version 5.01; GraphPad Software Inc., La Jolla, CA, USA). Thereby, the Wilcoxon matched pairs test was applied to check for statistically significant differences between the ischemia-affected areas and the corresponding contralateral areas, whereas the Mann-Whitney *U* test was used for time-dependent gene expression in the ischemia-affected areas. In general, values are given as means ± standard deviation (*M*/SD). A *p* value of < 0.05 was considered as statistically significant (*p* < 0.05 (*), *p* < 0.01 (**)). Panels on histochemical-based micrographs were prepared with Microsoft PowerPoint (version 2015; Microsoft Corp., Redmond, WA, USA). If necessary, brightness and contrast of images were slightly adjusted without creating or deleting signals.

## Results

### Qualitative Cellular Alterations Related to Focal Cerebral Ischemia

**Fig. 1:**
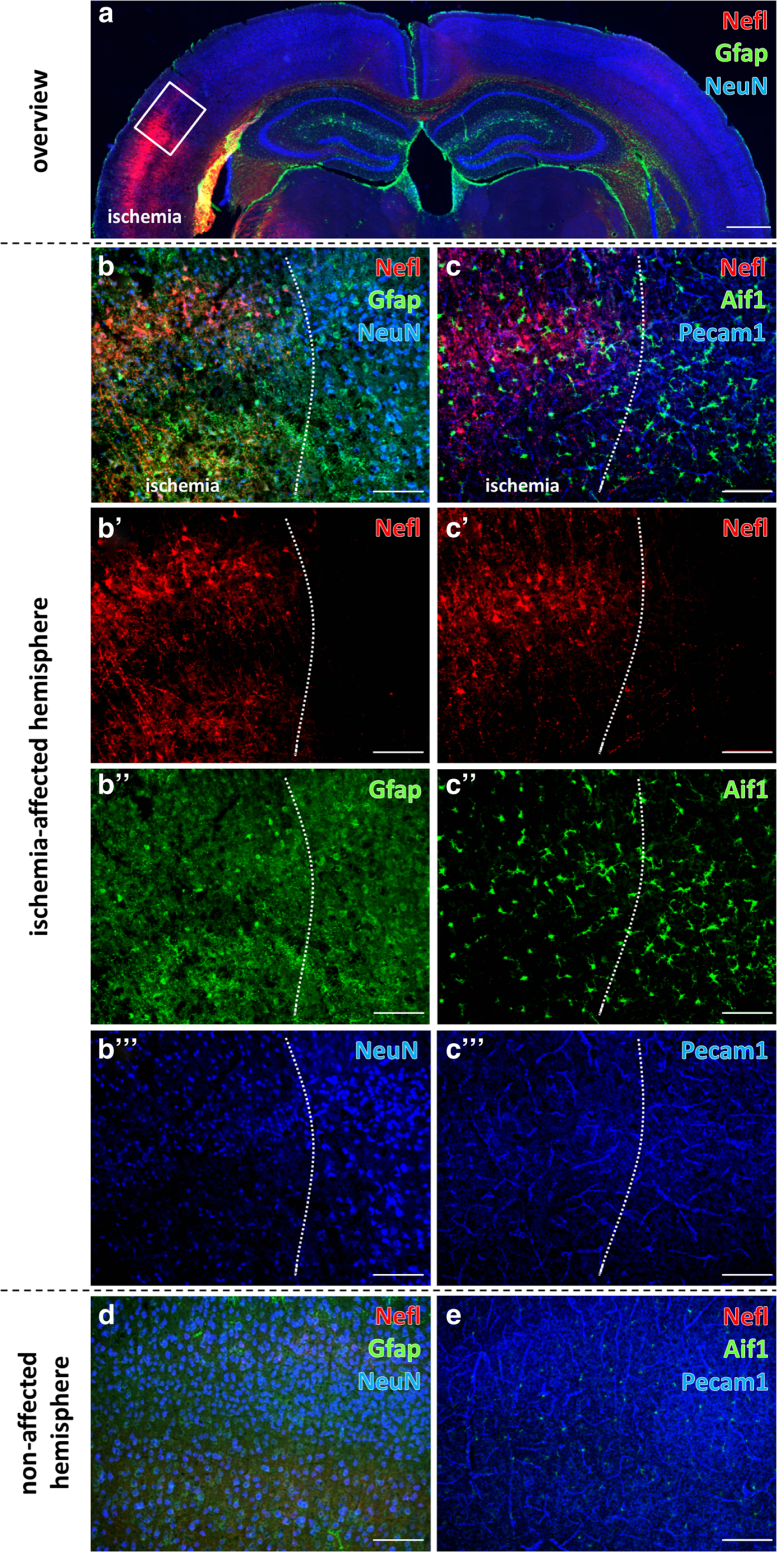
Immunolabeling of the neurofilament-L (Nefl) combined with astroglial Gfap and neuronal NeuN as well as microglia/macrophages (Aif1) and endothelial Pecam1 in the neocortex of mice affected by 24 h of focal ischemia. Figure 1**a** provides a general overview of the infarcted neocortex in the superior part of a forebrain section. Here, the ischemic area is delineated by an increased immunosignal of Nefl and a concomitantly decreased immunosignal of NeuN. The rectangle indicates the ischemic border zone, shown in **b** and **c** at higher magnification to explore regional arrangements. Here, a significant increase of Nefl-immunoreactivity (red, **b**′, **c**′), apparently fragmented astrocytes illustrated by immunolabeling of Gfap (green, **b**″), decreased NeuN immunosignals (**b**‴), ameboid Aif1-positive microglia/macrophages (**c**″), and a slightly decreased Pecam1 immunoreactivity (**c**‴) are visible in the ischemic area. Corresponding contralateral control areas show homogeneous distribution patterns for NeuN and Gfap (**d**), an only weak immunosignal of Nefl (**d**, **e**) and an evenly distributed Pecam1 immunoreactivity. Scale bars: **a** 300 μm; **b**–**e** 100 μm

To address ischemia-induced cellular reactions in a qualitative and spatial manner, triple immunofluorescence staining of the neurofilament light chain (Nefl) as part of the neuronal cytoskeleton, the astrocytic glial fibrillary acidic protein (Gfap), and the neuronal marker for neuronal nuclei (NeuN) were applied to mouse forebrain sections subjected to 24 h of ischemia (Fig. 1). Here, ischemia-affected areas in the neocortex were identified by a strong increase of Nefl immunoreactivity as described by Härtig et al. [54] and a simultaneous decrease of the NeuN immunosignal. In this area, the physiological appearance of distinct Gfap-stained astrocytic cellular processes was lost (Fig. 1A, B). Analogous analyses of ischemia-affected striatal areas provided similar staining patterns of Nefl and NeuN (data not shown). As expected, the neocortex of the contralateral, non-affected hemisphere was characterized by a consistent distribution of NeuN and Gfap together with a merely weak Nefl immunosignal (Fig. 1D). For analyses on microglia/macrophages, the allograft inflammatory factor 1 (Aif1), also known as ionized calcium-binding adapter molecule 1 (Iba1), was applied in combination with an endothelial marker, the platelet endothelial cell adhesion molecule (Pecam1 as cluster of differentiation 31, CD31), in the ischemia-affected neocortex. Here, an ameboid appearance of Aif1-positive microglia/macrophages was noted in ischemic tissue, as demarked by an increased immunosignal for Nefl, together with a slightly decreased Pecam1 immunoreactivity (Fig. 1C). Similar staining patterns of Nefl, Aif1, and Pecam1 were observed in the ischemia-affected striatum (data not shown). On the contralateral hemisphere, a naturally strong Pecam1 immunoreactivity was detectable together with a few and delicate appearing microglia/macrophages and—as expected—a weak immunosignal for Nefl (Fig. 1E).

### mRNA Levels for Structural Elements Depending on Duration of Ischemia and Brain Region

**Fig. 2:**
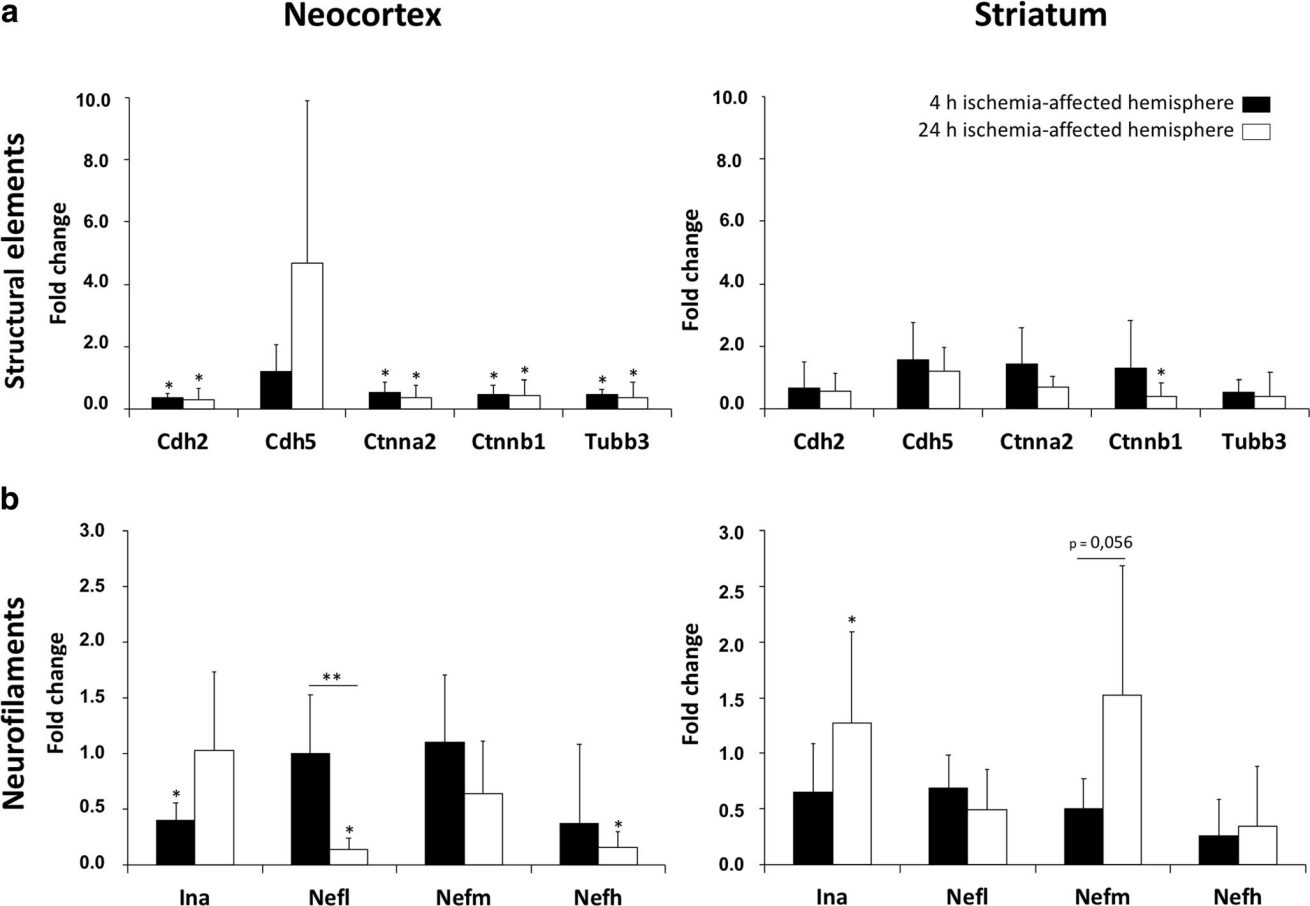
Comparative quantification of mRNA levels obtained from real-time PCR analyses of structural elements (**a**) and neurofilaments (**b**) after 4 and 24 h of ischemia in the neocortex and the striatum. Data are given as fold changes, calculated by an inter-hemispheric relation considering mRNA levels from the ischemic hemisphere (neocortex and striatum) and the corresponding contralateral, i.e., non-affected, regions. Bars represent means and added lines the standard deviations. Significance levels at the top of added lines are related to the inter-hemispheric comparison, while added horizontal lines with significance levels are related to potential temporal changes. *p < 0.05; **p < 0.01; sample size: *n* = 5 for each time point

To explore the transcriptional response of selected genes for structural elements (Table 1), expression analyses with qRT-PCR were performed in the neocortex and the striatum (Fig. 2a). Four hours after ischemia onset, neocortical mRNA levels of all analyzed genes except VE-cadherin (*Cdh5*) were significantly down-regulated by about 50%, whereat the fold change ranged between 0.55 for α-catenin 2 (*Ctnna2*) and 0.38 for N-cadherin (*Cdh2*). Notably, these mRNA levels remained significantly decreased until 24 h after ischemia onset. However, the mRNA level of *Cdh5* appeared to be numerically increased after 24 h of ischemia, but failed to change significantly in ischemic tissue. Compared to mRNA levels in the neocortex, the respective expression levels in the striatum differed markedly. Here, only the mRNA level of β-catenin 1 (*Ctnnb1*) was significantly diminished after 24 h of ischemia. Remarkably, by comparing the temporal profile of mRNA levels of related structural elements, i.e., from 4 and 24 h after ischemia, no significant time-dependent changes were revealed.

### Morphological Characteristics of Structural Elements in the Ischemia-Affected Neocortex

**Fig. 3:**
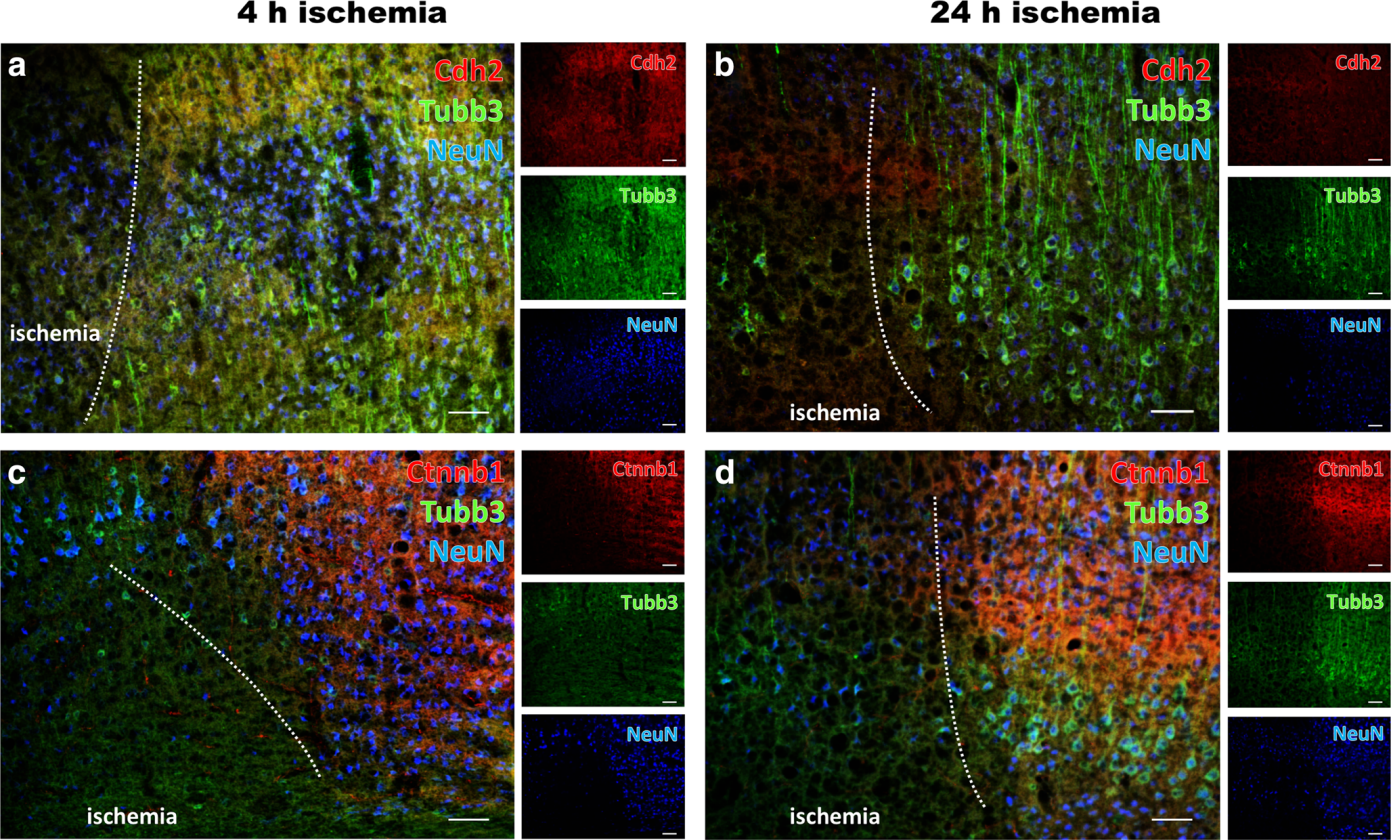
Immunofluorescence labeling of structural elements in the neocortex affected by 4 and 24 h of focal ischemia. Diminished immunosignals of N-cadherin (Cdh2, red, **a**, **b**) and β-catenin 1 (Ctnnb1, red, **c**, **d**) as well as βIII-tubulin (Tubb3, green, **a**–**d**), together with the neuronal marker neuronal nuclei (NeuN, blue, **a**–**d**), indicate morphological changes in terms of degenerated structural elements due to ischemia. While considering the duration of ischemia, the degree of altered immunosignals for structural elements indicates a more severe affection towards the later time point, i.e., 24 h of ischemia (**b**, **d**). Scale bars: **a**–**d** 100 μm

To explore the spatiotemporal pattern of structural elements associated with the observed changes in mRNA levels, triple immunofluorescence labeling was applied while focusing on structural elements of the ischemia-affected neocortex (Fig. 3). In detail, βIII-tubulin (Tubb3) was combined with N-cadherin (Cdh2) or β-catenin 1 (Ctnnb1), and neuronal nuclei (NeuN) as neuronal marker.

Thereby, the neuronal affection was visualized by time-dependently diminished immunosignals of NeuN and Tubb3 in ischemic areas (Fig. 3a–d). On the cellular level, 4 h of ischemia appeared to be associated with morphologically changed but still existing cellular elements positive for Tubb3 (Fig. 3a, c), while after 24 h of ischemia the cellular-associated continuity of Tubb3-positive structures was widely lost (Fig. 3b, d). Concerning Ctnnb1, a comparable pattern was found as the respective immunosignal was significantly decreased in areas of ischemic affection visualized by a decreased number of NeuN-positive neurons (Fig. 3c, d). However, the time-dependent effect appeared much less intensive when compared to Tubb3, while a significant reduction of the Ctnnb1 immunosignal was already visible at 4 h after ischemia (Fig. 3c) and continued towards 24 h of ischemia (Fig. 3d). With respect to Cdh2, a weak affection was detectable 4 and 24 h after ischemia in terms of a decreasing immunosignal in ischemic areas, slightly emphasizing the later time point (Fig. 3a, b).

### Changes in Neurofilament mRNA Levels Referring to Brain Region and Duration of Ischemia

Extending own data on the transcriptional response of internexin α (*Ina*), neurofilament light chain (*Nefl*), neurofilament medium chain (*Nefm*), neurofilament heavy chain (*Nefh*), at 24 h after ischemia onset [27], a subset of analyses focused on potential time-dependent reactions with reference to an earlier time point, i.e., 4 h of ischemia (Fig. 2b).

Thereby, the earlier time point exhibited a significant decrease of *Ina* mRNA level (fold change 0.39, *p* < 0.05) in the ischemia-affected neocortex when compared to the contralateral hemisphere, whereas the expression of the other neurofilaments remained unchanged. However, the striatal transcriptional responses of all neurofilament markers after 4 h of ischemia were found to be gradually diminished without statistical significance. The direct comparison of neurofilament mRNA levels between 4 and 24 h of ischemia revealed significantly changed mRNA levels of *Nefl* in the neocortex, whereas a down-regulation of about 86% towards 24 h of ischemia was found. By investigating the time-dependent striatal alterations, it became obvious that none of the analyzed neurofilament-associated genes provided a significant change from 4 to 24 h after ischemia onset, but *Nefm* mRNA levels were found to increase gradually towards 24 h of ischemia, closely missing statistical significance.

### Altered Gene Expression of NVU Components and Associated Extracellular Structures

**Fig. 4:**
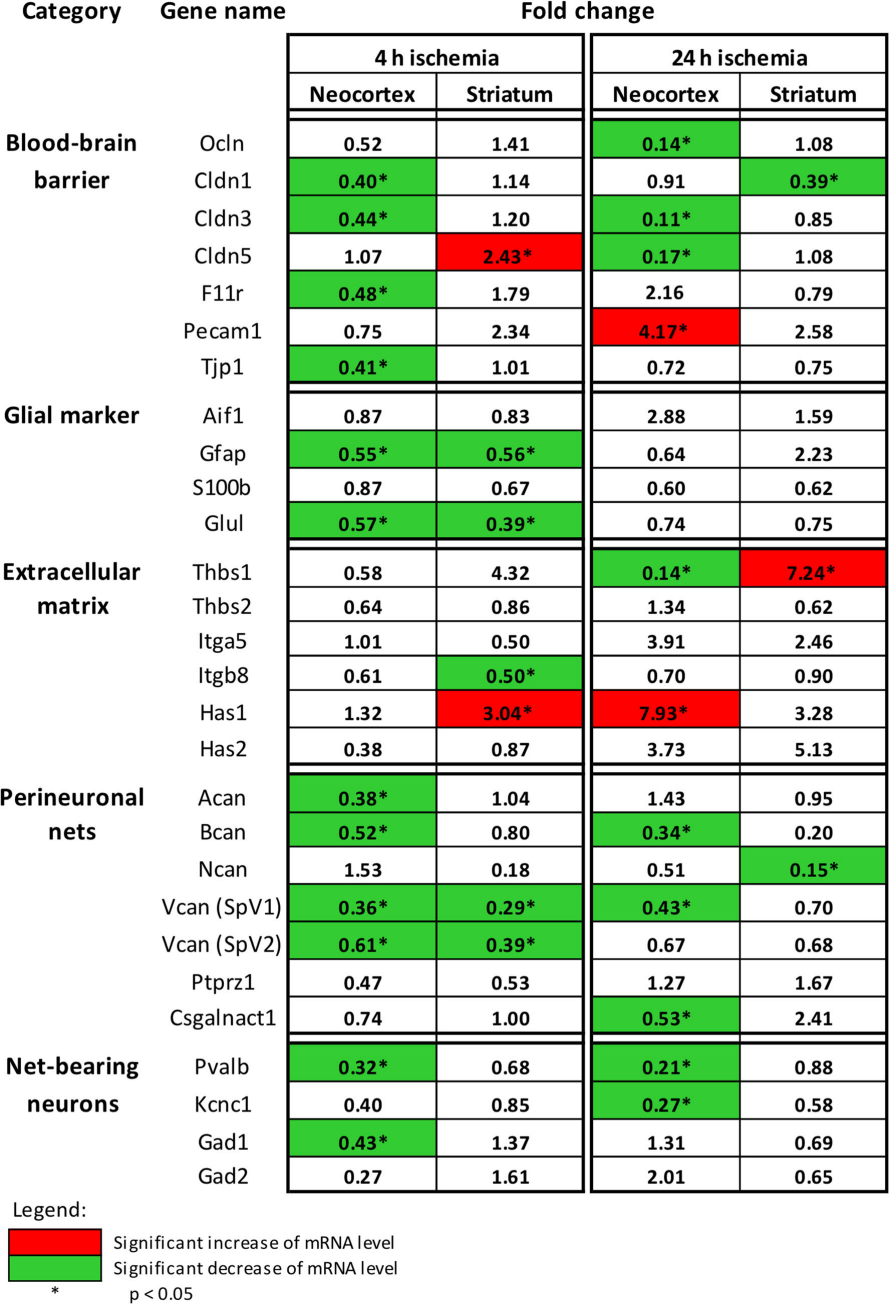
Changes in mRNA levels revealed by real-time PCR analyses of different markers of the blood-brain barrier, the neurovascular unit, and associated extracellular matrix constituents 4 and 24 h after ischemia onset in the neocortex and the striatum. The given values represent fold changes, calculated as the ratio of the mRNA levels between the ischemic hemisphere (neocortex and striatum) and the corresponding contralateral, i.e., non-affected, regions. Thereby, significant changes are color-coded: A green background represents a down-regulation, a red background stands for an up-regulation, whereas a white background indicates no relevant changes. *p < 0.05; sample size: *n* = 5 for each time point

To address the concomitant transcriptional response of NVU components and the associated extracellular matrix, mRNA levels of previously selected and clustered genes (Table 1) were assessed at 4 and 24 h after ischemia onset in the neocortex and the striatum*.* For easier survey, the targeted genes were categorized according to the localization of their transcription products within the NVU and ECM, respectively (Fig. 4, abbreviations listed in Table 4).

**Table 4:** Abbreviations

Category	Gene ID	Name
Structural elements	*Cdh2*	N-cadherin
	*Cdh5*	VE-cadherin
	*Ctnna2*	α-catenin 2
	*Ctnnb1*	β-catenin 1
	*Tubb3*	βIII-tubulin
Neurofilaments	*Ina*	Internexin α
	*Nefh*	Neurofilament heavy chain
	*Nefl*	Neurofilament light chain
	*Nefm*	Neurofilament medium chain
Blood-brain barrier	*Cldn1*	Claudin-1
	*Cldn3*	Claudin-3
	*Cldn5*	Claudin-5
	*F11r (Jam1)*	F11-receptor
	*Ocln*	Occludin
	*Pecam1 (CD31)*	Platelet endothelial cell adhesion molecule 1
	*Tjp1*	Tight junction protein1
Glial markers	*Aif1 (Iba)*	Allograft inflammatory factor 1
	*Gfap*	Glial fibrillary acidic protein
	*Glul (GS)*	Glutamine synthetase
	*S100b*	S100β
Extracellular matrix	*Has1*	Hyaluronan synthase 1
	*Has2*	Hyaluronan synthase 2
	*Itga5*	Integrin-α5
	*Itgb8*	Integrin-β8
	*Thsb1*	Thrombospondin-1
	*Thsb2*	Thrombospondin-2
Perineuronal nets	*Acan*	Aggrecan
	*Bcan*	Brevican
	*Csgalnatc1*	Chondroitin sulfate N-acetylgalactosaminyltransferase 1
	*Ncan*	Neurocan
	*Ptprz1*	Phosphacan
	*Vcan*	Versican
Net-bearing neurons	*Gad1*	Glutamate decarboxylase 1
	*Gad2*	Glutamate decarboxylase 2
	*Kcnc1 (Kv3.1b)*	Voltage-gated potassium channel subfamily C member 1
	*Pvalb*	Parvalbumin
Houskeeping genes	*Actb*	β-actin
	*Gapdh*	Glyceraldehyde 3-phosphate dehydrogenase

Roughly summarized, the mRNA levels of the analyzed genes exhibited a rather inhomogeneous pattern, while most of the addressed genes did not reach a statistically significant change due to ischemia. But importantly, of the 112 listed mRNA levels, 25.0% were significantly diminished in ischemic areas, whereas only 3.6% of the mRNA levels were significantly increased. Accordingly, the majority of the listed target genes (applying for 68.8% of the listed mRNA levels, independent from time point and region) exhibited a more than 10% numerically decreased mRNA level in ischemia-affected tissue compared with the respective controls. Only a minority of 28.5% of the listed target genes (independent from time point and region) exhibited a more than 10% numerically increased mRNA level. Remarkably, in relation to the analyzed brain region, 69.7% of the significantly altered mRNA levels were detected in the neocortex. In detail, claudin-5 (*Clnd5*) and *Pecam1* as BBB-associated genes, as well as thrombospondin-1 (*Thbs1*) and hyaluronan synthase 1 (*Has1*) as genes related to the ECM exhibited significantly increased mRNA levels throughout the applied time points and brain regions. In terms of categorization, a lot of genes associated with glial markers, PNs, or net-bearing neurons exhibited no significant changes, but at the same time there were some markers that provided significantly decreased mRNA levels as for instance glutamine synthetase (*Glul*), parvalbumin (*Pvalb*), glutamate decarboxylase 1 (*Gad1*) as well as aggrecan (*Acan*), brevican (*Bcan*), neurocan (*Ncan*), and the splice variants 1 and 2 of versican (*Vcan*)*.*

With regard to time-dependent changes, some genes exhibited a remarkable profile. Notably, neocortical mRNA levels for integrin α-5 (*Itga5*) provided an at least numerically relevant increase from 4 to 24 h of ischemia, while *Has1* was found to increase significantly during this period. In line, striatal mRNA levels for *Itga5* and for the splice variants 1 of versican (*Vcan*) were also found to increase from 4 to 24 h of ischemia.

### Ischemic Consequences Regarding Protein Levels of Pecam1 and Has1

**Fig. 5:**
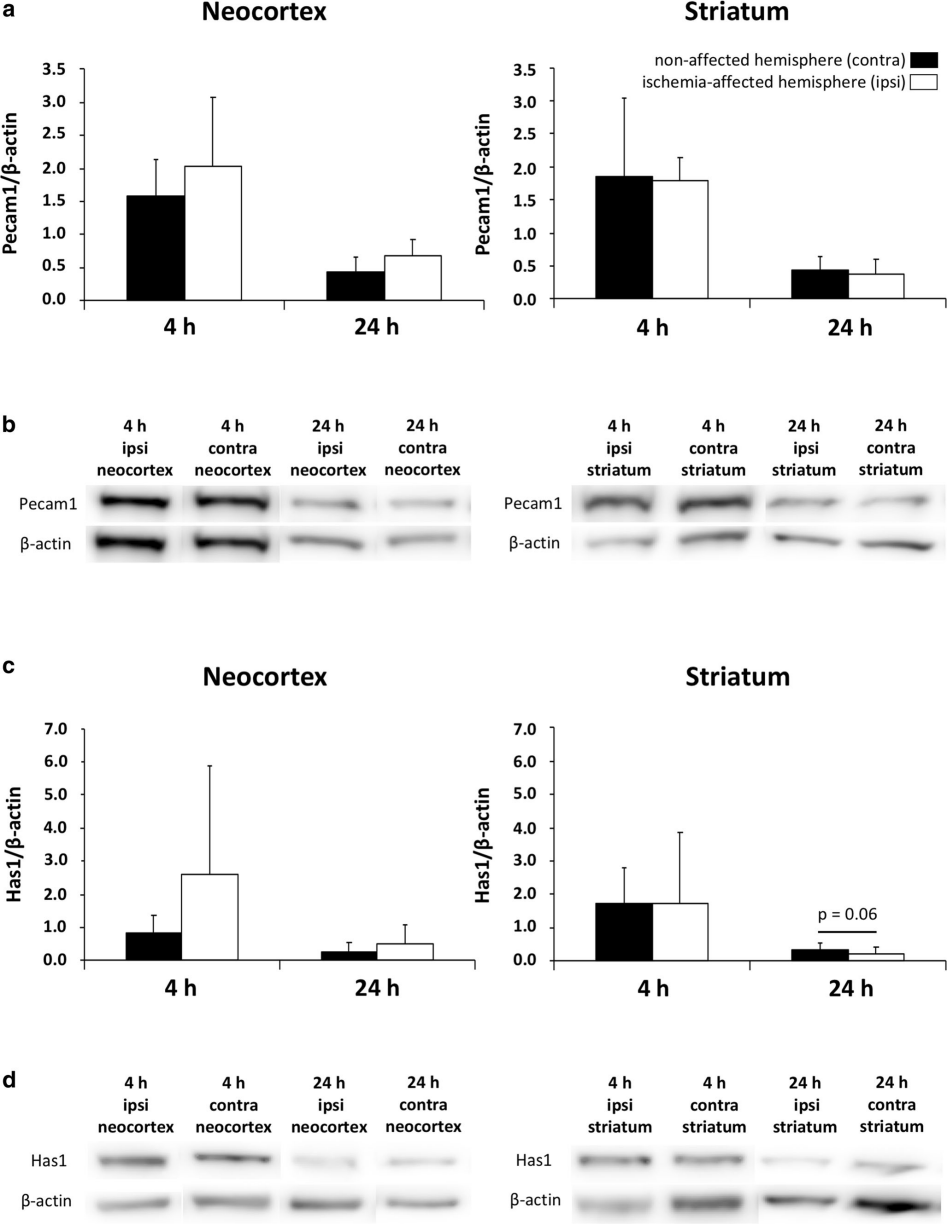
Comparative quantifications of protein levels of selected blood-brain barrier- and extracellular matrix-related markers obtained from Western blot analyses after 4 and 24 h of ischemia in the neocortex and the striatum. **a** The Pecam1 protein levels normalized by β-actin remain statistically unaltered after ischemia, but tend to increase in the ischemia-affected neocortex 4 and 24 h after ischemia. **c** The neocortical and striatal protein levels of hyaluronan synthase 1 (Has1) normalized by β-actin similarly show a non-significant increase in the ischemia-affected neocortex 4 and 24 h after ischemia. However, in the striatum the protein level tends to decrease 24 h after ischemia, barely missing statistical significance. **b**, **d** Representative protein expression bands of Pecam1 (**b**) and Has1 (**d**) with corresponding bands of β-actin (**b**, **d**) in samples from neocortex and striatum 4 and 24 h after ischemia. Bars represent means and added lines the standard deviations. Sample size: *n* = 5 for each time point

Given the observation of variously changed mRNA levels due to ischemia, a subset of analyses focused on the respective protein levels as detectable by Western blot analysis (Fig. 5). However, among the exemplarily selected BBB-associated platelet endothelial cell adhesion molecule 1 (Pecam1) and the ECM-related hyaluronan synthase 1 (Has1), the protein levels did not change significantly following ischemia while considering neocortical and striatal areas as well as the duration of ischemia, i.e., 4 and 24 h. Remarkably, at 24 h after ischemia, a numerical decrease of the protein level for Has1 was noted in the striatum, closely missing statistical significance. Further trends were noted for Pcam1 in the neocortex, while both 4 and 24 h of ischemia resulted in a numerical increase of the protein level (Fig. 5a). A comparable pattern was also found for Has1 in the neocortex with a gradual increase of the respective protein levels 4 and 24 after ischemia (Fig. 5c).

### Morphological and Region-Specific Features of Glial Markers Following Ischemia

**Fig. 6:**
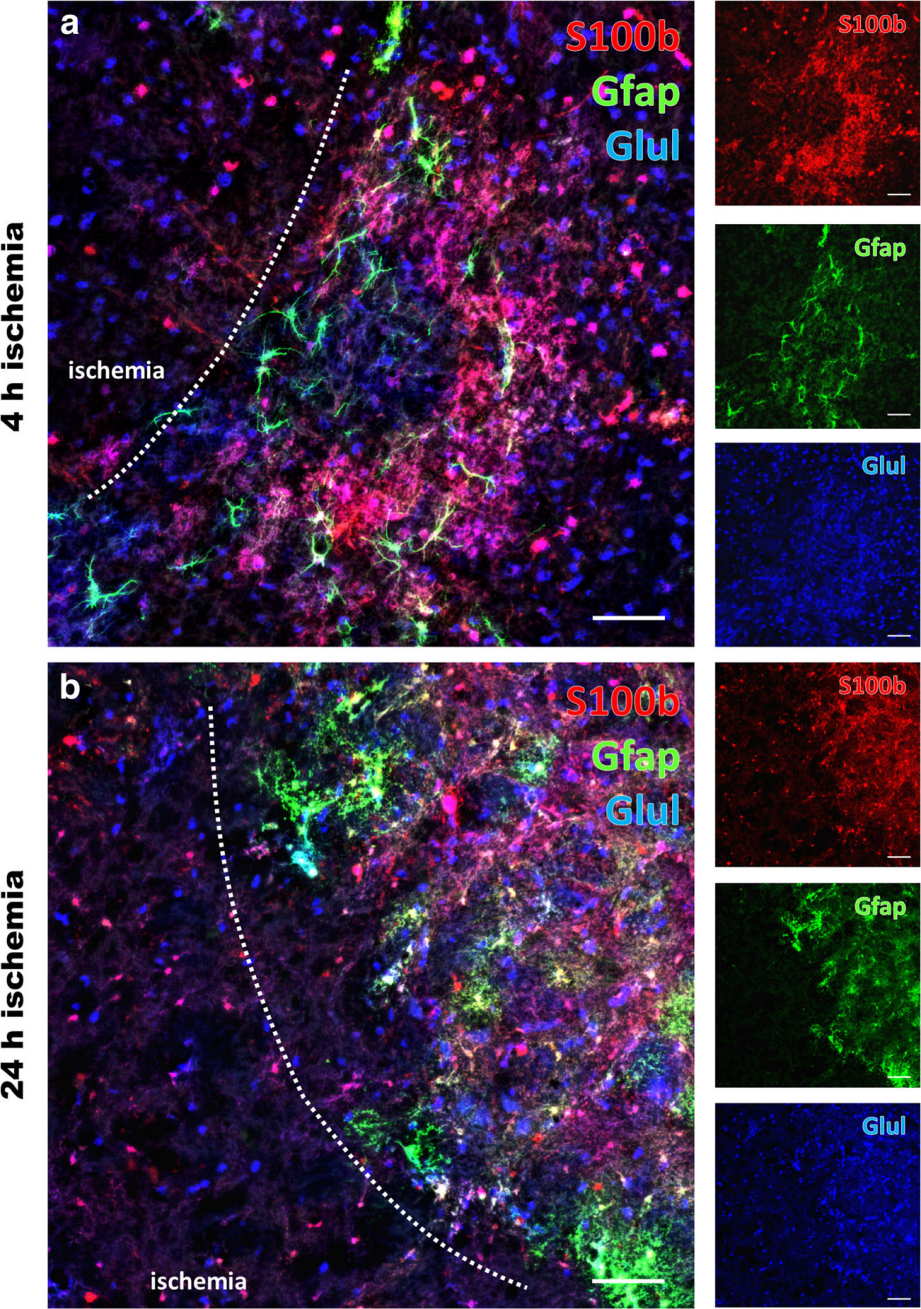
Glial alterations following 4 and 24 h of focal cerebral ischemia. Multiple immunofluorescence labeling applied to the ischemia-affected striatum reveals significantly altered signals of the astroglial markers S100β (S100b, red, **a**, **b**) and Gfap (green, **a**, **b**) as well as glutamine synthetase (Glul, blue, **a**, **b**) decreasing towards ischemic regions. When considering potential time-dependent effects, the degree of affection seems to be pronounced towards the later time point, i.e., 24 h after ischemia (**b**). Remarkably, directly at the ischemic border zone, slightly increased immunosignals become visible for Gfap and S100b with a carpet-like appearance. Scale bars: **a**–**d** 100 μm

As transcriptional analyses have revealed significant alterations of the glial fibrillary acidic protein (*Gfap*) and the enzyme glutamine synthetase (*Glul*), representing genes associated with astrocytes and the glutamine synthesis, further multiple immunofluorescence labeling was applied to capture morphological features of these markers after 4 and 24 h of ischemia. In detail, Gfap and Glul were combined with S100β (S100b) as a further astroglial marker (Fig. 6), illustrating drastically changed immunosignals of all three markers in ischemic areas. In detail, the area of clear ischemia-related affection was demarked by a decrease of the immunosignal for S100b and Gfap and—to a much lesser degree—also Glul, accompanied with a degradation of the cellular formations in this region (Fig. 6a, b). Remarkably, at the border zone towards the ischemic tissue, a slightly increased immunosignal of Gfap and even clearer S100b was visible together with a carpet-like appearance of these markers (Fig. 6a). This feature seemed to differ with respect to the duration of ischemia, emphasizing the later time point, i.e., 24 h of ischemia (Fig. 6b).

### Regional Characterization of Perineuronal Nets and Net-Bearing Neurons

**Fig. 7:**
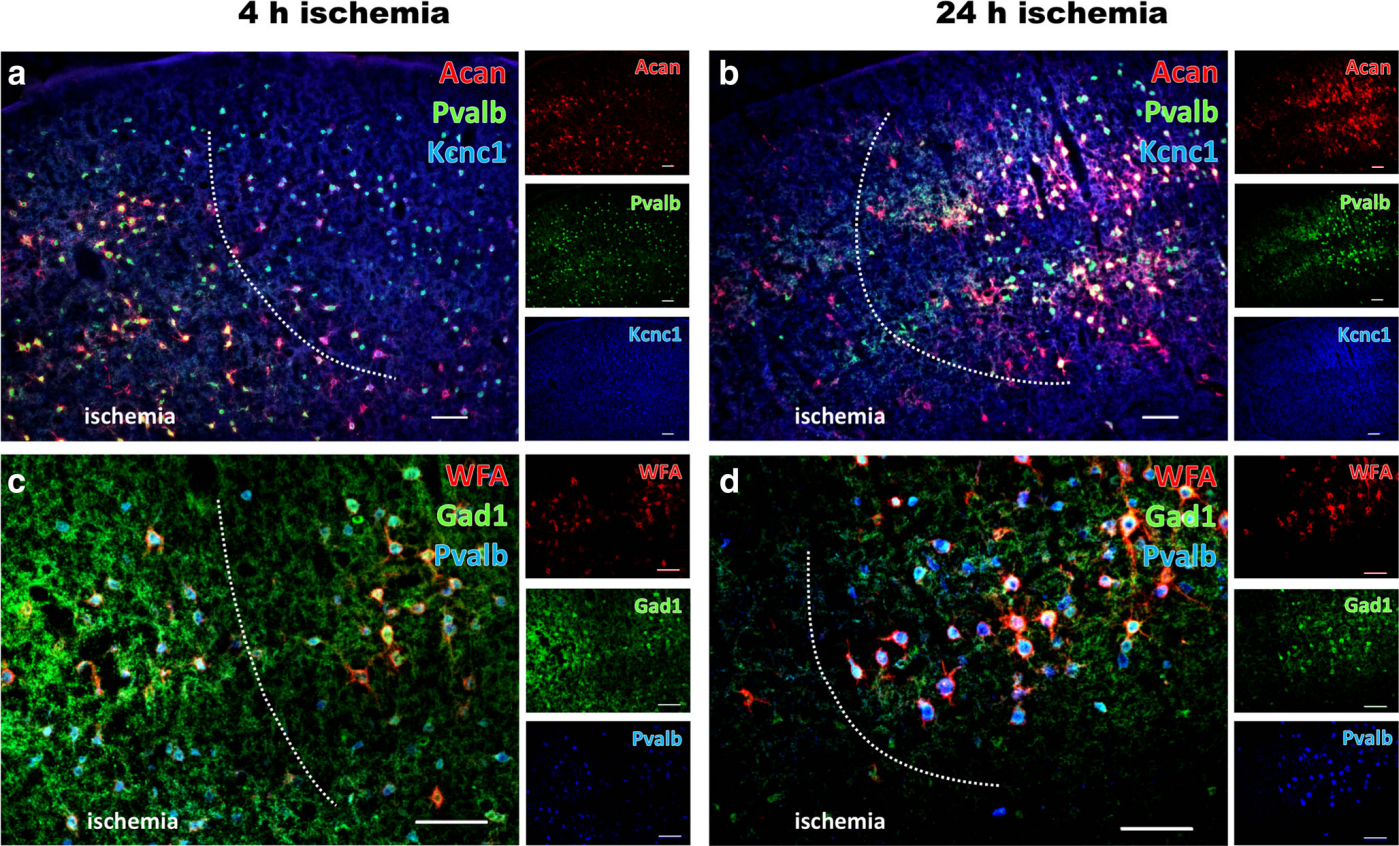
Alterations of perineuronal nets and net-bearing neurons in the neocortex affected by 4 and 24 h of focal ischemia. Multiple immunofluorescence labeling of aggrecan (Acan, red, **a**, **b**), combined with the visualization of parvalbumin (Pvalb, green, **a**, **b**) and the voltage-gated potassium channel subfamily C member 1 (Kcnc1, blue, **a**, **b**) as markers for a large subset of net-bearing neurons indicate significantly decreased immunosignals for Acan and Pvalb after 24 h of ischemia (**b**), while the respective signals after 4 h of ischemia (**a**) appear largely stable. Additional time-dependent changes are revealed by triple fluorescence labeling of enzyme glutamate decarboxylase 1 (Gad1, green, **c**, **d**), Pvalb (blue, **c**, **d**) and binding sites for the lectin *Wisteria floribunda* agglutinin (WFA, red, **c**, **d**). Notably, the labelling of all 3 markers is strongly decreased strongly decreased in the ischemic zone after 24 h of ischemia (**d**). Although to a much lesser degree, a degradation of net-like structures becomes already visible after 4 h of ischemia (**c**) in terms of a weakened signal for WFA in ischemic areas. Scale bars: **a**–**d** 100 μm

To further correlate the observed transcriptional changes with alterations on the morphological level, multiple immunofluo\rescence labeling was performed including the potassium voltage-gated channel subfamily C member 1 (Kcnc1) and parvalbumin (Pvalb) in numerous net-ensheathed neurons, as well as the classical net component aggrecan (Acan). With the intention to consider potential time-dependent effects, this approach included both durations of ischemia (Fig. 7a, b). Thereby, 4 h of ischemia was associated with a weakly diminished immunosignal of Kcnc1 in the ischemia-affected region (Fig. 7a), whereas Pvalb remained stable and also the Acan immunoreactivity robustly maintained towards the ischemic region. In contrast, 24 h of ischemia resulted in a considerably decreased immunosignal of both Acan and Pvalb (Fig. 7b).

Since the prepended transcriptional explorations further identified the glutamate decarboxylase 1 (*Gad1*) as significantly altered marker, additional multiple fluorescence analyses were applied involving the immunodetection of Gad1 and Pvalb together withlectin-histochemistry of *Wisteria floribunda* agglutinin (WFA) for the visualization of N-acetylgalactosamine residues from N- and O-glycans (Fig. 7c, d). Here, the lectin staining revealed a degradation of net-like structures that became visible already after 4 h of ischemia (Fig. 7c) and resulted in a nearly complete decomposition of these structures after 24 h of ischemia (Fig. 7d). Comparable patterns with especially a clear time-dependent affection were found for Gad1 and Pvalb (Fig. 7c, d).

## Discussion

Using a multi-methodological approach to capture transcriptional reactions and morphological features of cellular and non-cellular components within the NVU and the associated ECM, this study was intended to provide further details on pathophysiological processes following ischemic stroke. Thereby, qRT-PCR and Western blot analyses as well as immunofluorescence labeling were carried out on brain tissues subjected to 4 and 24 h of focal ischemia. These time points were chosen to consider the naturally given time-dependent course of ischemia evolution [47]. In contrast to earlier attempts on the identification of appropriate biomarkers by mainly analyzing blood-sourced and thus peripheral RNA as indicators for processes occurring in the brain, this study analyzed a variety of selected markers directly within ischemic brain tissue and separately for changes related to the neocortex and striatum. Consequently, this gene expression study is the first report trying to conflate the transcriptional response of NVU- and ECM-related genes with morphological features of the associated cellular and non-cellular constituents, specified for different brain areas and time points.

### Transcriptional Responses Related to Focal Cerebral Ischemia

The present study revealed changes of mRNA levels as early as 4 h after ischemia onset and in a region-specific fashion, which highlights the analyzed elements as being involved in the very early and thus therapeutic relevant phase after stroke. This observation further emphasizes the need for a time- and region-dependent characterization after focal cerebral ischemia.

Among the analyzed genes, significantly altered mRNA levels were predominantly localized in the ischemic neocortex. This finding might reflect an enhanced vulnerability of neocortical areas to the ischemic stimulus, as compared to striatal areas. Otherwise, a relevant portion of striatal tissue naturally consists of afferent and efferent fiber tracts, where protein biosynthesis usually does not take place. However, the physiological distribution of neuronal somata, visualized by NeuN labeling of the contralateral and thus non-affected hemisphere in Fig. 1, shows a more intense neuronal immunosignal in the neocortex than the adjacent subcortical regions. Therefore, it could be hypothesized that neuron-related changes of mRNA level are more probable to reach statistical significance in the neocortex, representing an area of homogeneously distributed neuronal somata. This might explain, why from the genes related to PNs or net-bearing neurons, the majority of the significantly decreased mRNA levels occurred in the neocortex. Concerning time-dependent aspects of transcriptional responses, there was surprisingly no statistically significant differences when examining the mRNA data set as a whole. Only 4 of 37 analyzed genes exhibited time-dependent changes, independently of their localization.

Overall, this widespread dataset is suitable to give an impression of transcriptional responses due to ischemia, here simplified by stating an ischemia-related decrease or increase of mRNA levels with reference to the individual contralateral and thus non-affected hemisphere. Generally, the task of interpreting mRNA data in the setting of stroke is rather challenging, since gene expression interpretations are usually based on the assumption that the measured mRNA levels are being transcribed into the respective proteins, thereby affecting cellular functions and ultimately cellular morphology. In the center of the infarct, neurons and astrocytes are known to cease protein biosynthesis, as soon as the cerebral blood flow decreases to 50% [55]. Nevertheless, attempts of an interpretation will be given here for selected mRNA results with the hypothesis that protein biosynthesis in at least peripheral brain areas affected by ischemia, i.e., penumbral regions, is still functioning.

By analyzing neurofilaments, earlier reports showed decreasing protein levels for Nefl and Nefh as investigated by immunolabeling and blotting techniques after acute stimuli to the brain, as for instance traumatic brain injury and experimental stroke [56–59]. Consistent data originated from quantitative mRNA analyses demonstrated a reduction of the mRNA levels for *Nefl* and *Nefh* at 24 h after the induction of experimental stroke in rodents [28]. Referring to time-dependent effects, the recent results thus extended the perspective of ischemic consequences to neurofilaments towards the early phase of stroke. Thereby, the expression of *Nefl* was found to persist at the basic level at 4 h after ischemia induction with the exception of *Ina* that displayed a significant decrease already at this early stage. Thus, neurofilaments and among them especially *Ina*, *Nefl*, and *Nefh* can be considered as strong markers during the first day after ischemia. These and other cytoskeletal elements, such as the microtubule protein βIII-tubulin, are essential for the maintenance of neuronal stability [60] and thus might be candidates for future neuroprotective approaches. The here consistently measured decrease of *Tubb3* mRNA following 4 and 24 h of ischemia strengthened earlier reports [61], emphasizing the pivotal role of cytoskeletal elements during ischemia formation.

A loss of the neuronal adherence junction molecule N-cadherin (*Cdh2*) due to ischemia, as might be interpreted from the measured diminished *Cdh2* mRNA level, can cause neuroepithelial and thereby structural damage [62]. Together with the also observed ischemia-derived reduction of β-catenin 1 (*Ctnnb1*), which is interacting with N-cadherin, these changes might critically affect cell-cell adhesion processes [63, 64]. However, the here observed down-regulation of *Ctnnb1* mRNA at 4 and 24 h after ischemia can also be interpreted by its function as the main downstream mediator of the canonical Wnt signaling pathway. This pathway is known to promote development, proliferation, and regeneration processes [65], to influence the expression of targeted genes, like the here investigated versican (*Vcan*) and claudin-1 (*Cldn1*), in which a reduced level of β-catenin 1 results in a down-regulation of *Vcan* and *Clnd1* expression [66, 67]. Consequently, this cascade might represent a potential target for therapeutic approaches by influencing neuro-regenerative processes [68, 69]. Another cell adhesion molecule belonging to the group of catenins is the neuronal form of α-catenin 2 (*Ctnna2*). This protein is assumed to play a major role in the folding and arrangement of cortical layers, and was shown to be affected in experimental stroke studies [70, 71]. In the present study, a significant decrease of the *Ctnna2* expression was observed at 4 and 24 h of ischemia, suggesting that such fundamental processes become critical affected during the early phase of stroke.

From a more functional perspective involving the BBB, experiments with β-catenin-deficient mice revealed that as a consequence a down-regulation of *Cldn1* and *Cldn3*, but not of *Cldn5*, leads to a BBB dysfunction [72]. Accordingly, the present study revealed a significant decrease of mRNA levels for *Cldn1* and *Cldn3* after ischemia, which might be provoked by the simultaneously occurring decline of *Ctnnb1*. *Cldn5*, however, exhibited an inconsistent pattern, yielding both increased and decreased mRNA levels depending on the time point and the brain region. This finding suggests complex processes or dependencies such as the type of ischemia, which is supported by earlier investigations showing a decreased *Cldn5* gene expression after experimental stroke with a reperfusion scenario [73, 74]. Further, the present data indicated a time critical reaction of *Cldn5* in the neocortex with a stable condition at 4 h and a significantly down-regulated mRNA level towards 24 h after stroke induction. In a slightly different manner but also supporting the perspective of a temporal expression pattern, Liu et al. noted a decreased *Cldn5* expression that starts already at 1 h with a maximum decline at 8 h after experimental ischemia to the rat brain [75]. Other mRNA levels of genes associated with tight junctions, such as occludin (*Ocln*), zonula occludens-1, encoded by the tight junction protein1 gene (*Tjp1*), as well as the before mentioned *Cldn1* and *Cldn3*, are either significantly decreased due to ischemia or unchanged, depending on time point and brain region. These data are in accordance with other studies, showing decreased mRNA levels of *Tjp1* and *Ocln* 120 h after focal cerebral ischemia [74]. The down-regulation of the above-mentioned mRNA levels might ultimately lead to a decrease of the respective proteins, thereby endangering local hemostasis and BBB function [76, 77]. This perspective is supported by an earlier study demonstrating that a loss of *Ocln* from cerebral microvessels after ischemia is associated with massive BBB leakage [78, 79].

Closely associated with the integrity of the BBB is the maintenance of endothelial structures. Among these, the endothelial transmembrane receptor molecule CD31, encoded by the *Pecam1* gene, is also known to have immunomodulatory properties and is thus critical involved angiogenesis [80, 81]. The present study revealed that the gene expression of *Pecam1* was non-significantly decreased at 4 h, but significantly increased in the neocortex affected by 24 h of ischemia. Consistently, for a longer observational period, Guo et al. reported an up-regulation of the CD31 expression around the infarcted area after permanent focal cerebral in the rat [82]. These findings suggest that long-lasting and the BBB integrity critically involving endothelial changes already started within the first day after ischemia onset.

By focusing on the transcriptional response of glial elements following ischemia, a subset of analyses addressed the gene related to the allograft inflammatory factor 1 (*Aif1*) associated with microglia as well as the genes related to the glial fibrillary acidic protein (*Gfap*), S100β (*S100b*), and the glutamine synthetase (*Glul*) associated with astrocytes. In a previous study, microglia was identified to react already 3.5 h after experimental focal cerebral ischemia lasting up to 7 days as visualized by immunolabeling of Iba1, encoded by *Aif1* [83]. In the present study, however, the *Aif1* gene expression failed to provide a significant reaction after 4 or 24 h of ischemia. In contrast, the mRNA level of neocortical and striatal *Gfap* decreased as early as 4 h after ischemia. Consequently, this astroglial protein captured by mRNA analyses can be considered as a sensitive marker for astrocyte reaction during early stages of ischemia. From a more functional perspective, the astrocytic enzyme glutamine synthetase, encoded by the gene *Glul*, converts glutamate into glutamine and is thus critically involved during stroke progression, because glutamate represents a central mediator for excitotoxity [47]. Likewise, in the present study, the *Glul* gene was found to decrease significantly at 4 h after ischemia induction.

The concept of the NVU also includes the adjacent ECM with PNs, known to influence a variety of cellular functions as well as cellular integrity and neural plasticity [8, 25]. As a constituent of the ECM, integrins are located at the cell surface serving as receptors that facilitate cell-cell or cell-matrix interactions [84]. In the present study, mRNA levels of *Itga5*—encoding the α-subunit of α5β1 integrin—failed to provide a significant reaction with reference to the non-affected hemisphere but were found to increase from 4 to 24 h of ischemia. This observation might be explained by the fact that this part of the ECM is involved in processes of post-ischemic angiogenesis, which is supported by earlier data that linked α5β1 integrin with the up-regulation of the vascular endothelial growth factor expression, known to be necessary for formation of new vessels [85]. In contrast to *Itga5*, the mRNA level of *Has1*, encoding for a hyaluronan synthase isoform, which synthesizes hyaluronan [86], was found to increase significantly in the present study. This is in line with a previous study showing an up-regulated protein level of Has1 under ischemic conditions [87], while the current data specified the temporal profile towards the 4- and 24-h time point after ischemia. Genes related to PNs were also associated with the NVU and thus included in the present qRT-PCR analyses. Here, the measured gene expression of the lecticans aggrecan (*Acan*), brevican (*Bcan*), and neurocan (*Ncan*), both splice variants of versican (*Vcan)* as well as chondroitin sulfate N-acetylgalactosaminyltransferase 1 (*Csgalnact1*), as components of the CSPGs that are widely expressed in the normal central nervous system [88, 89], reacted in a decreasing manner in the ischemia-affected striatum and neocortex. These mRNA data are in line with previous investigations describing an ischemia-related degradation of PN components [14, 90, 91]. The reaction of other PN-associated proteins like ionic channels or transporters might be relevant in conjunction with ischemic consequences of the NVU and related ECM. Thereby, the detected down-regulated mRNA levels for markers of net bearing GABAergic neurons, e.g. *Pvalb* and *Kcnc1* (also known as Kv3.1b) might reflect the ischemia-caused significant affection of the GABAergic system, as an earlier study discussed that the loss of inhibitory neurons for Pvalb and Kv3.1b could impair the GABAergic wiring in the cortical microcircuitry [92]. In a second way, this affection might lead to a dysfunction of net-associated parvalbumin in terms of a more plastic state [93]. Further, the here measured decrease of *Gad1* expression, representing a key enzyme (glutamate decarboxylase 1) for GABA synthesis, might reflect dysfunctional GABAergic neurons ultimately leading to an impaired GABA synthesis [94, 95].

### Morphological Features and Protein Production Along with Transcriptional Responses

Bringing together the findings from qRT-PCR analyses with the morphological features visualized by immunofluorescence labeling, a considerable proportion of markers exhibited a consistent way of reaction, simplified as either increases or decreases due to the ischemic stimulus. For example, the structural elements βIII-tubulin (Tubb3), N-cadherin (Cdh2), and β-catenin 1 (Ctnnb1) were found to react with decreased mRNA levels and concomitantly diminished immunosignals indicating relevant morphological changes in terms of cellular degeneration. Furthermore, consistent data were obtained for glial markers (Gfap, Glul) as well as PN constituents (Acan) and net-bearing neurons (Pvalb and Kcnc1). With respect to the protein levels, Western blot analyses failed to provide significant changes of selected markers in the setting of ischemia, but yielded—at least in terms of a trend—increased protein levels of Pecam1 and Has1 in the ischemia-affected neocortex that is in good accordance with the qRT-PCR results.

Apart from these conclusive findings, an inconsistent pattern became further evident for neurofilaments as the present study—in terms of a completion of previous investigations related to the time point of 24 h after ischemia onset [27]—also failed to demonstrate an increased mRNA level for any of the neurofilaments investigated after 4 h of ischemia, which might be expected from the consistently detectable increased immunosignal of Nefl 24 h of ischemia. Potential reasons were extensively discussed earlier [27]. However, the present study further raises questions on the occurrence of a significantly decreasing mRNA level for *Gad1* 4 h after ischemia, which could not be detected on the immunofluorescence analysis at this early stage, but on the later time point. Further, the relatively strong increases of the mRNA level for *Pecam1* after 24 h of ischemia was not seen in respective immunofluorescence analyses.

These data on partially conflicting observations between mRNA levels and morphological changes, as visualized by immunofluorescence labeling, reinforce the still challenging task to interpret up- and down-regulated mRNA levels. However, while considering the existing weakness of immunofluorescence- and thus antibody-based techniques that harbor the risk of an increased antibody reaction due to an antigen fragmentation resulting in an increased amount of antibody binding sites, changes in fluorescence signals need to be interpreted with caution too. On the protein level, the present findings might also support previous observations of a critically reduced protein biosynthesis caused by a relevant reduction of the cerebral blood flow [55]. This perspective is further strengthened by the performed subset of Western blot analyses that yielded non-significant results regarding the ischemia- and non-affected hemisphere. In case of accumulating evidence, this limitation would prospectively impede further research applying biochemical techniques on directly affected brain tissues.

### Methodological Considerations

The present study has some limitations: First, although efforts have been made to include a variety of genes and to consider more than a single time point for explorations, experiments were restricted to changes resulting from 4 and 24 h of ischemia. Therefore, further explorations of the time course of selected genes including both very early and more delayed periods after the ischemic event seem to be essential. Nevertheless, the present approach considered the early and a more delayed phase of stroke, both being relevant for current and future therapeutic interventions. Second, this study was restricted to a single model of permanent middle cerebral artery occlusion, which was chosen because of its translational relevance by mimicking the relevant proportion of patients who suffer from large vessel occlusion in the absence of therapeutic options. However, since the time-window for vessel re-opening strategies after acute ischemic stroke was recently extended [96], future analyses should also explore transcriptional responses after transient focal cerebral ischemia. Third, to correctly compare all the analyzed genes specified for different time points and brain areas, it was necessary to collectively measure all probes in one single PCR run. Therefore, only a limited number of animals could be included, inhibiting more robust statistical calculations due to an increase of the sample size. Fourth, not all genes investigated by qRT-PCR are available as markers feasible for immunohistochemistry. Although efforts have been made to include at least one candidate of structural elements, neurofilaments, glial, and endothelial structures as well as constituents of the ECM and their PNs into the histochemical analyses, newly generated antibodies might allow a more precise investigation of the mRNA morphology interrelation in the future.

## Conclusions

Despite the given limitations, the present study for the first time provides a comprehensive overview of transcriptional responses from cellular and non-cellular elements within the ischemia-affected NVU and ECM, in addition to immunofluorescence and Western blot analyses. On the transcriptional level as explored by qRT-PCR, simultaneous changes of a large set of NVU- and ECM-related genes were observed as early as 4 h after experimental focal cerebral ischemia, while statistically significant alterations exhibited a mainly decreasing direction. Comparative analyses at the transcriptional and morphological levels indicated that several structural elements, as for instance βIII-tubulin, N-cadherin, and β-catenin 1, as well as glial markers and PN constituents displayed concomitant morphological alterations in terms of degenerations on the cellular and non-cellular level. The revealed expression pattern illustrates the complexity of affected cell populations within the NVU and associated non-cellular structures after stroke. These insights might help to focus future research with a pathophysiological orientation, as for example by exploring the role of structural elements and neurofilaments within the NVU in more detail. Such information might prospectively help to facilitate novel neuroprotective strategies beyond the traditional single target perspective.
